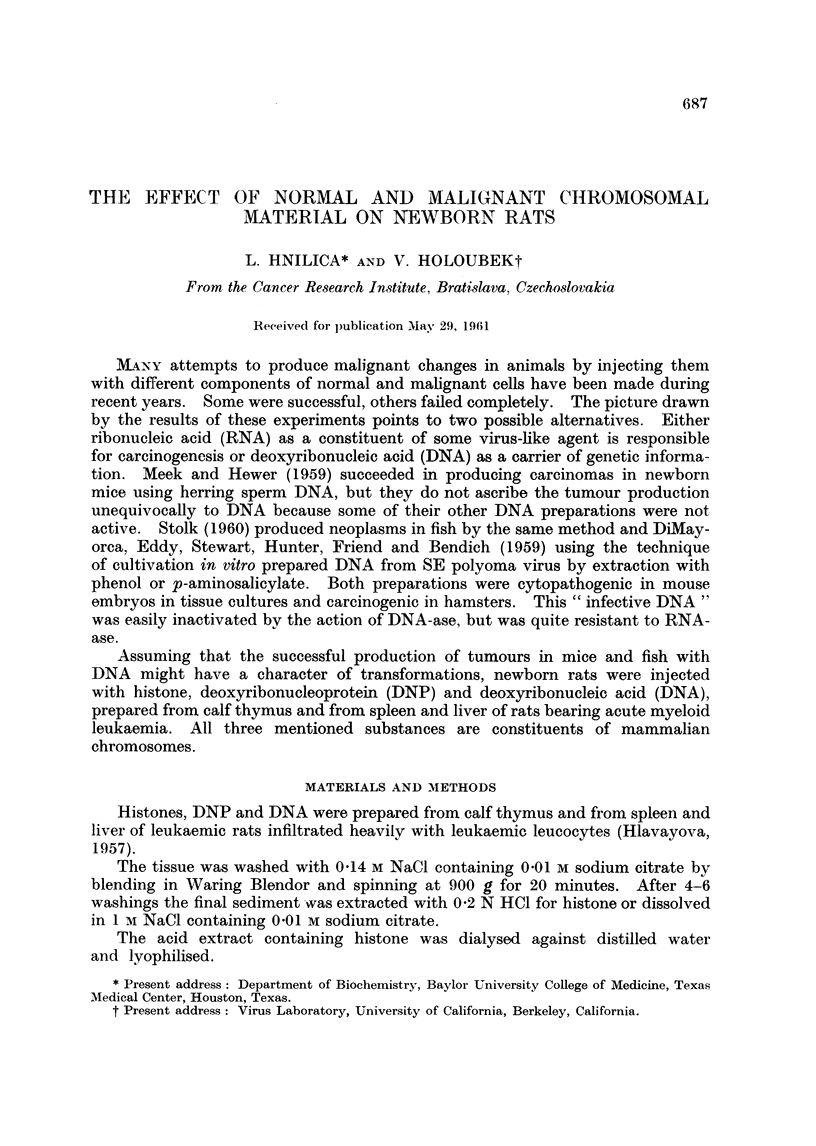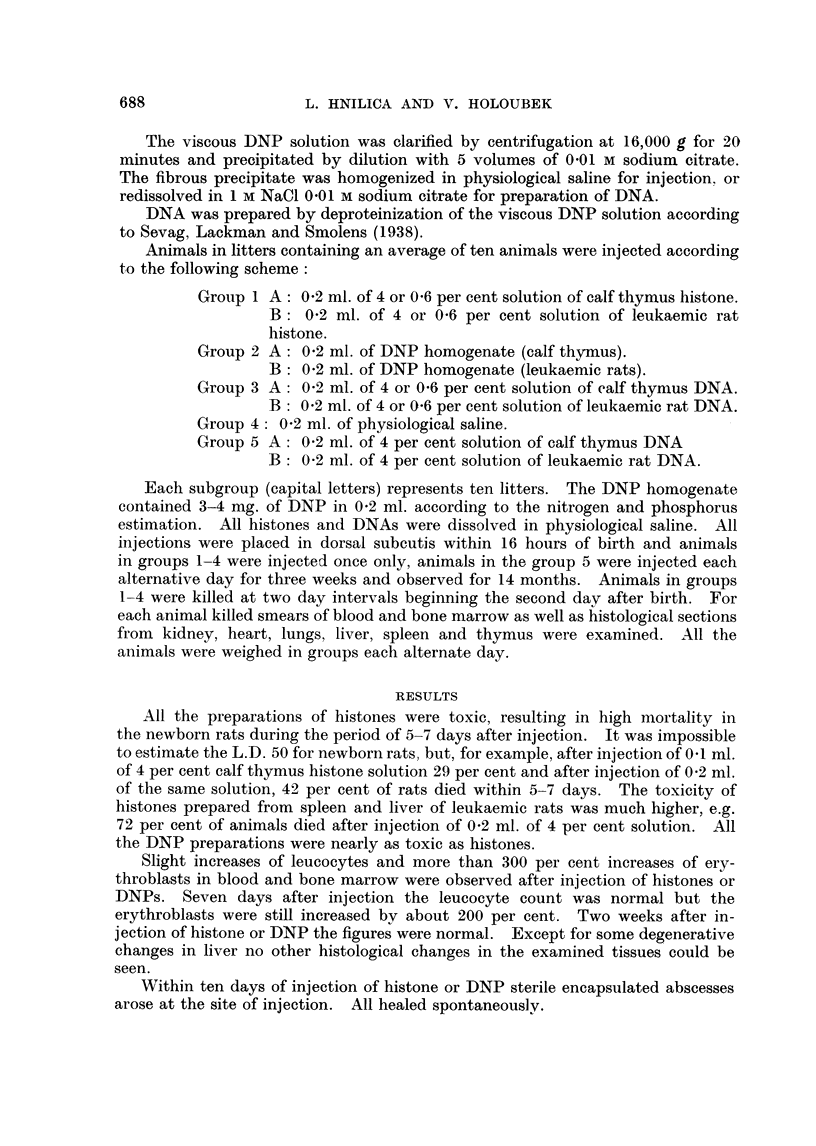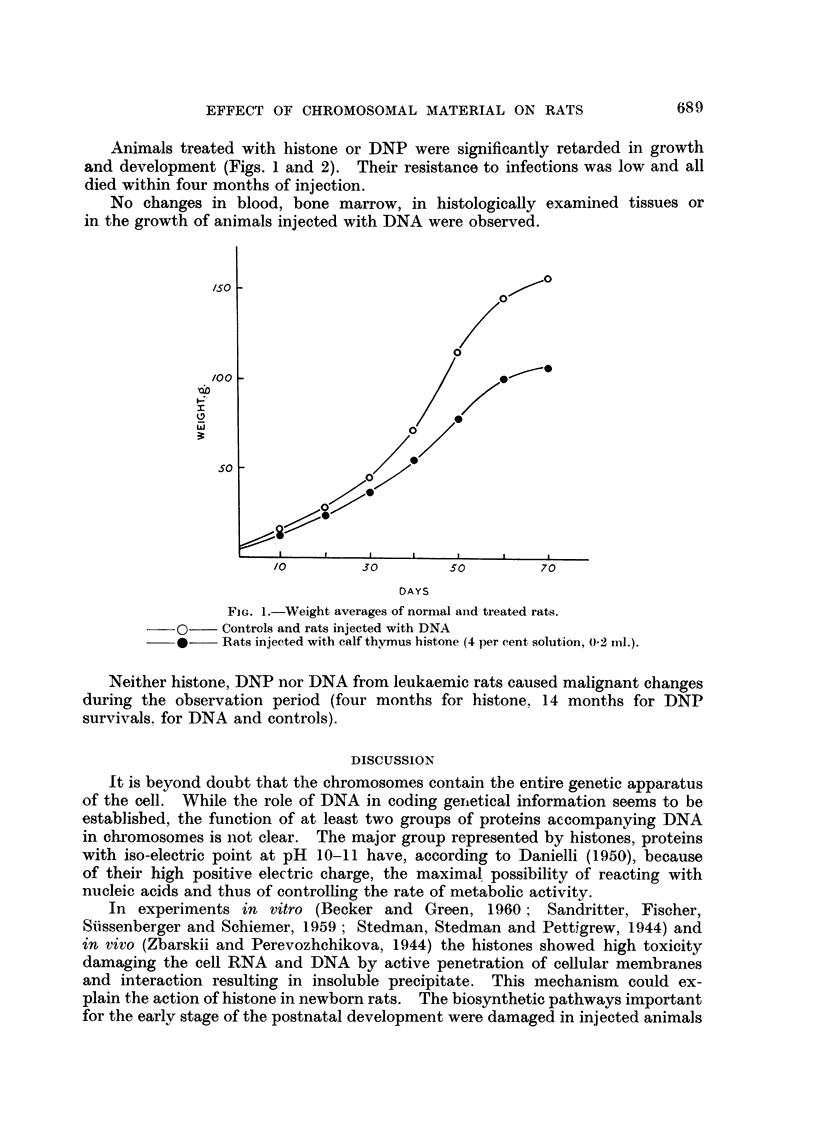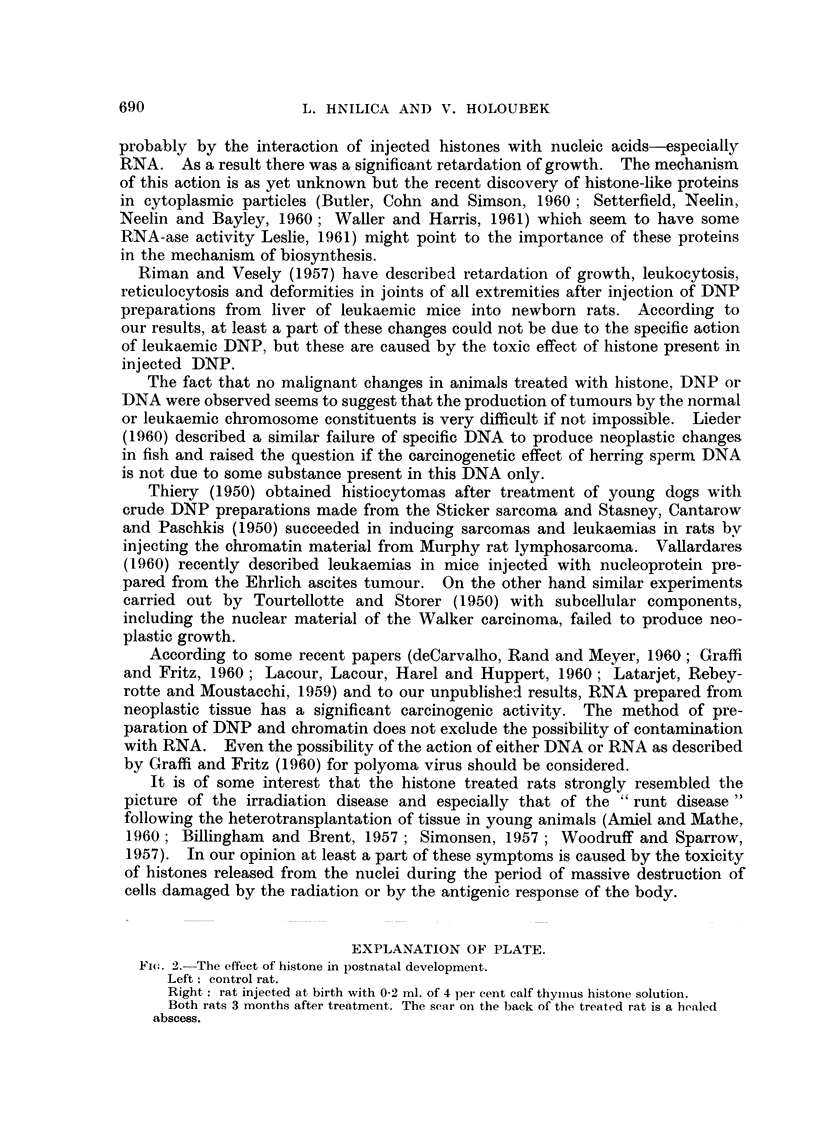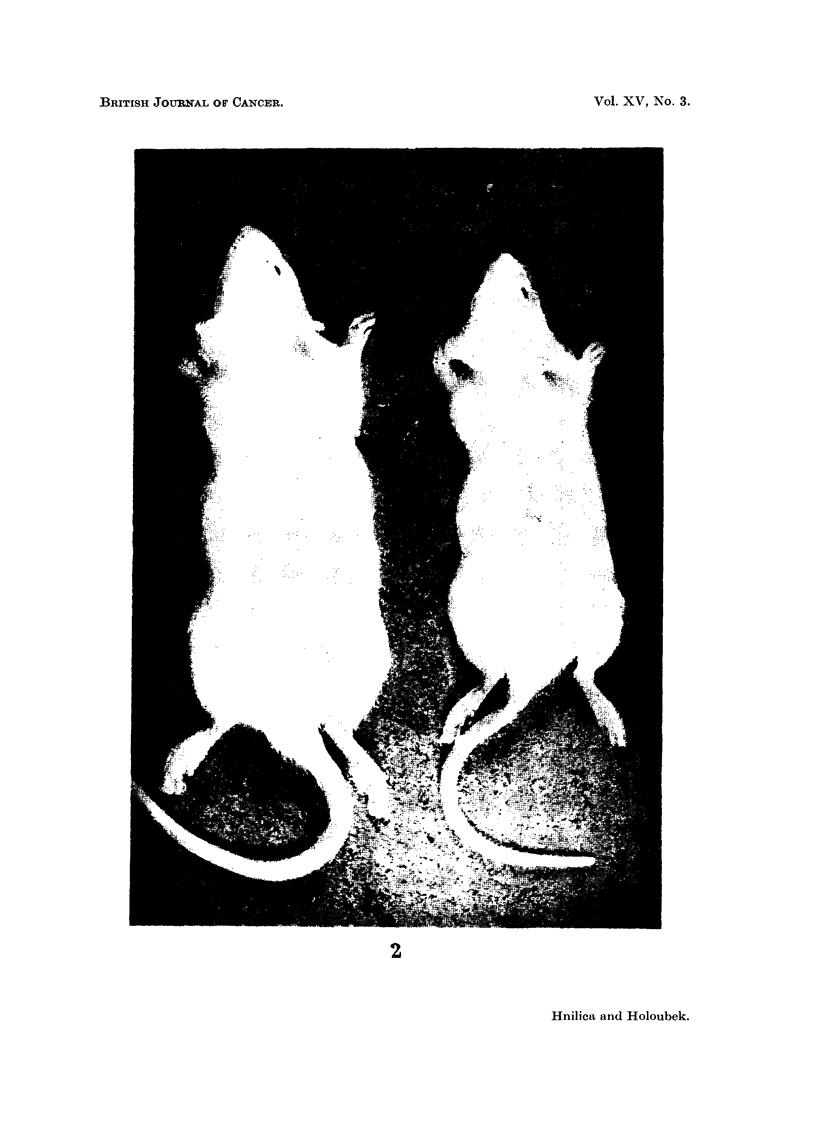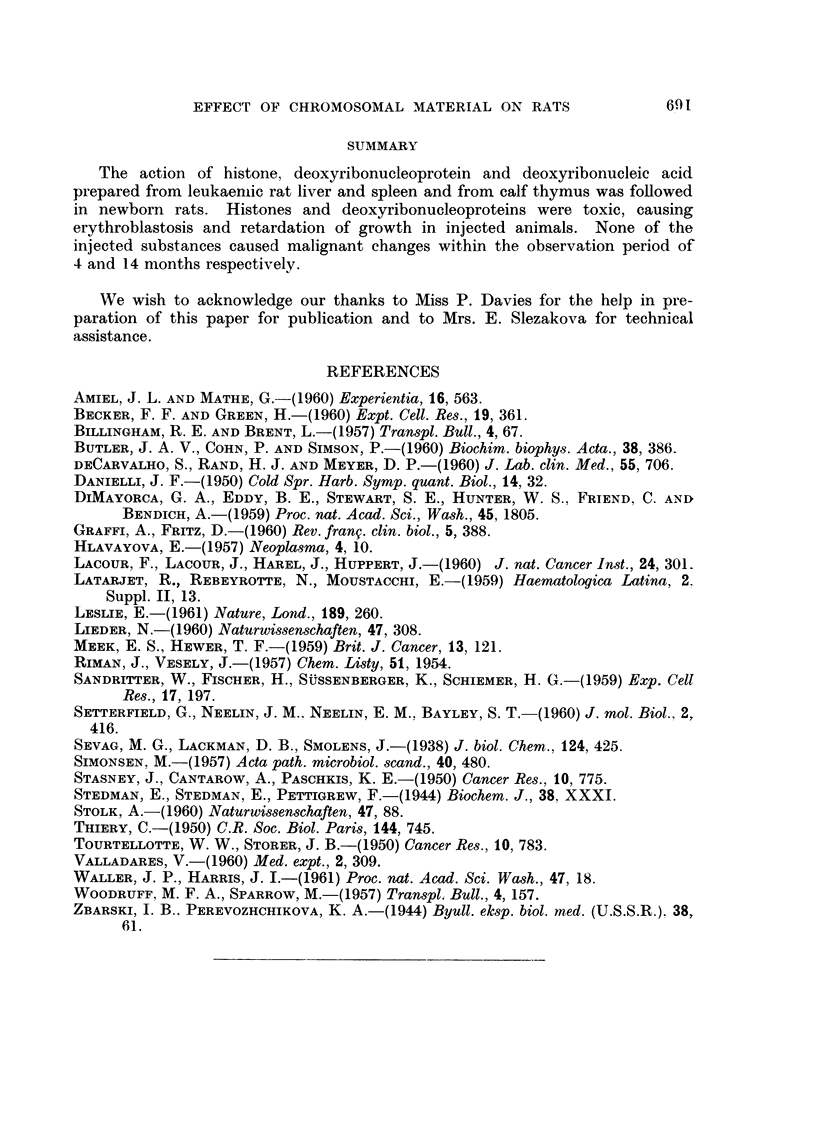# The Effect of Normal and Malignant Chromosomal Material on Newborn Rats

**DOI:** 10.1038/bjc.1961.78

**Published:** 1961-09

**Authors:** L. Hnilica, V. Holoubek

## Abstract

**Images:**


### THE EFFECT OF NORMAL AND MALIGNANT CHROMOSOMAL MATERIAL ON NEWBORN RATS

L. HNILICA* AND V. HOLOUBEK†

*From the Cancer Research Institute, Bratislava, Czechoslovakia*

Received for publication May 29, 1961

MANY attempts to produce malignant changes in animals by injecting them with different components of normal and malignant cells have been made during recent years. Some were successful, others failed completely. The picture drawn by the results of these experiments points to two possible alternatives. Either ribonucleic acid (RNA) as a constituent of some virus-like agent is responsible for carcinogenesis or deoxyribonucleic acid (DNA) as a carrier of genetic information. Meek and Hewer (1959) succeeded in producing carcinomas in newborn mice using herring sperm DNA, but they do not ascribe the tumour production unequivocally to DNA because some of their other DNA preparations were not active. Stolk (1960) produced neoplasms in fish by the same method and DiMayorca, Eddy, Stewart, Hunter, Friend and Bendich (1959) using the technique of cultivation *in vitro* prepared DNA from SE polyoma virus by extraction with phenol or *p*-aminosalicylate. Both preparations were cytopathogenic in mouse embryos in tissue cultures and carcinogenic in hamsters. This " infective DNA " was easily inactivated by the action of DNA-ase, but was quite resistant to RNA-ase.

Assuming that the successful production of tumours in mice and fish with DNA might have a character of transformations, newborn rats were injected with histone, deoxyribonucleoprotein (DNP) and deoxyribonucleic acid (DNA), prepared from calf thymus and from spleen and liver of rats bearing acute myeloid leukaemia. All three mentioned substances are constituents of mammalian chromosomes.

### MATERIALS AND METHODS

Histones, DNP and DNA were prepared from calf thymus and from spleen and liver of leukaemic rats infiltrated heavily with leukaemic leucocytes (Hlavayova, 1957).

The tissue was washed with 0·14 M NaCl containing 0·01 M sodium citrate by blending in Waring Blendor and spinning at 900 *g* for 20 minutes. After 4–6 washings the final sediment was extracted with 0·2 N HCl for histone or dissolved in 1 M NaCl containing 0·01 M sodium citrate.

The acid extract containing histone was dialysed against distilled water and lyophilised.

* Present address : Department of Biochemistry, Baylor University College of Medicine, Texas Medical Center, Houston, Texas.

† Present address : Virus Laboratory, University of California, Berkeley, California.

The viscous DNP solution was clarified by centrifugation at 16,000 *g* for 20 minutes and precipitated by dilution with 5 volumes of 0·01 M sodium citrate. The fibrous precipitate was homogenized in physiological saline for injection, or redissolved in 1 M NaCl 0·01 M sodium citrate for preparation of DNA.

DNA was prepared by deproteinization of the viscous DNP solution according to Sevag, Lackman and Smolens (1938).

Animals in litters containing an average of ten animals were injected according to the following scheme :

Group 1 A : 0·2 ml. of 4 or 0·6 per cent solution of calf thymus histone.
B : 0·2 ml. of 4 or 0·6 per cent solution of leukaemic rat histone.
Group 2 A : 0·2 ml. of DNP homogenate (calf thymus).
B : 0·2 ml. of DNP homogenate (leukaemic rats).
Group 3 A : 0·2 ml. of 4 or 0·6 per cent solution of calf thymus DNA.
B : 0·2 ml. of 4 or 0·6 per cent solution of leukaemic rat DNA.
Group 4 : 0·2 ml. of physiological saline.
Group 5 A : 0·2 ml. of 4 per cent solution of calf thymus DNA
B : 0·2 ml. of 4 per cent solution of leukaemic rat DNA.

Each subgroup (capital letters) represents ten litters. The DNP homogenate contained 3–4 mg. of DNP in 0·2 ml. according to the nitrogen and phosphorus estimation. All histones and DNAs were dissolved in physiological saline. All injections were placed in dorsal subcutis within 16 hours of birth and animals in groups 1–4 were injected once only, animals in the group 5 were injected each alternative day for three weeks and observed for 14 months. Animals in groups 1–4 were killed at two day intervals beginning the second day after birth. For each animal killed smears of blood and bone marrow as well as histological sections from kidney, heart, lungs, liver, spleen and thymus were examined. All the animals were weighed in groups each alternate day.

### RESULTS

All the preparations of histones were toxic, resulting in high mortality in the newborn rats during the period of 5–7 days after injection. It was impossible to estimate the L.D. 50 for newborn rats, but, for example, after injection of 0·1 ml. of 4 per cent calf thymus histone solution 29 per cent and after injection of 0·2 ml. of the same solution, 42 per cent of rats died within 5–7 days. The toxicity of histones prepared from spleen and liver of leukaemic rats was much higher, e.g. 72 per cent of animals died after injection of 0·2 ml. of 4 per cent solution. All the DNP preparations were nearly as toxic as histones.

Slight increases of leucocytes and more than 300 per cent increases of erythroblasts in blood and bone marrow were observed after injection of histones or DNPs. Seven days after injection the leucocyte count was normal but the erythroblasts were still increased by about 200 per cent. Two weeks after injection of histone or DNP the figures were normal. Except for some degenerative changes in liver no other histological changes in the examined tissues could be seen.

Within ten days of injection of histone or DNP sterile encapsulated abscesses arose at the site of injection. All healed spontaneously.

Animals treated with histone or DNP were significantly retarded in growth and development (Figs. 1 and 2). Their resistance to infections was low and all died within four months of injection.

No changes in blood, bone marrow, in histologically examined tissues or in the growth of animals injected with DNA were observed.

FIG. 1.—Weight averages of normal and treated rats.
——○—— Controls and rats injected with DNA
——●—— Rats injected with calf thymus histone (4 per cent solution, 0·2 ml.).

Neither histone, DNP nor DNA from leukaemic rats caused malignant changes during the observation period (four months for histone, 14 months for DNP survivals, for DNA and controls).

### DISCUSSION

It is beyond doubt that the chromosomes contain the entire genetic apparatus of the cell. While the role of DNA in coding genetical information seems to be established, the function of at least two groups of proteins accompanying DNA in chromosomes is not clear. The major group represented by histones, proteins with iso-electric point at pH 10–11 have, according to Danielli (1950), because of their high positive electric charge, the maximal possibility of reacting with nucleic acids and thus of controlling the rate of metabolic activity.

In experiments *in vitro* (Becker and Green, 1960 ; Sandritter, Fischer, Süssenberger and Schiemer, 1959 ; Stedman, Stedman and Pettigrew, 1944) and *in vivo* (Zbarskii and Perevozhchikova, 1944) the histones showed high toxicity damaging the cell RNA and DNA by active penetration of cellular membranes and interaction resulting in insoluble precipitate. This mechanism could explain the action of histone in newborn rats. The biosynthetic pathways important for the early stage of the postnatal development were damaged in injected animals probably by the interaction of injected histones with nucleic acids—especially RNA. As a result there was a significant retardation of growth. The mechanism of this action is as yet unknown but the recent discovery of histone-like proteins in cytoplasmic particles (Butler, Cohn and Simson, 1960 ; Setterfield, Neelin, Neelin and Bayley, 1960 ; Waller and Harris, 1961) which seem to have some RNA-ase activity Leslie, 1961) might point to the importance of these proteins in the mechanism of biosynthesis.

Riman and Vesely (1957) have described retardation of growth, leukocytosis, reticulocytosis and deformities in joints of all extremities after injection of DNP preparations from liver of leukaemic mice into newborn rats. According to our results, at least a part of these changes could not be due to the specific action of leukaemic DNP, but these are caused by the toxic effect of histone present in injected DNP.

The fact that no malignant changes in animals treated with histone, DNP or DNA were observed seems to suggest that the production of tumours by the normal or leukaemic chromosome constituents is very difficult if not impossible. Lieder (1960) described a similar failure of specific DNA to produce neoplastic changes in fish and raised the question if the carcinogenetic effect of herring sperm DNA is not due to some substance present in this DNA only.

Thiery (1950) obtained histiocytomas after treatment of young dogs with crude DNP preparations made from the Sticker sarcoma and Stasney, Cantarow and Paschkis (1950) succeeded in inducing sarcomas and leukaemias in rats by injecting the chromatin material from Murphy rat lymphosarcoma. Vallardares (1960) recently described leukaemias in mice injected with nucleoprotein prepared from the Ehrlich ascites tumour. On the other hand similar experiments carried out by Tourtellotte and Storer (1950) with subcellular components, including the nuclear material of the Walker carcinoma, failed to produce neoplastic growth.

According to some recent papers (deCarvalho, Rand and Meyer, 1960 ; Graffi and Fritz, 1960 ; Lacour, Lacour, Harel and Huppert, 1960 ; Latarjet, Rebeyrotte and Moustacchi, 1959) and to our unpublished results, RNA prepared from neoplastic tissue has a significant carcinogenic activity. The method of preparation of DNP and chromatin does not exclude the possibility of contamination with RNA. Even the possibility of the action of either DNA or RNA as described by Graffi and Fritz (1960) for polyoma virus should be considered.

It is of some interest that the histone treated rats strongly resembled the picture of the irradiation disease and especially that of the " runt disease " following the heterotransplantation of tissue in young animals (Amiel and Mathe, 1960 ; Billingham and Brent, 1957 ; Simonsen, 1957 ; Woodruff and Sparrow, 1957). In our opinion at least a part of these symptoms is caused by the toxicity of histones released from the nuclei during the period of massive destruction of cells damaged by the radiation or by the antigenic response of the body.

### EXPLANATION OF PLATE.

FIG. 2.—The effect of histone in postnatal development.

Left : control rat.

Right : rat injected at birth with 0·2 ml. of 4 per cent calf thymus histone solution.

Both rats 3 months after treatment. The scar on the back of the treated rat is a healed abscess.

2

SUMMARY

The action of histone, deoxyribonucleoprotein and deoxyribonucleic acid prepared from leukaemic rat liver and spleen and from calf thymus was followed in newborn rats. Histones and deoxyribonucleoproteins were toxic, causing erythroblastosis and retardation of growth in injected animals. None of the injected substances caused malignant changes within the observation period of 4 and 14 months respectively.

We wish to acknowledge our thanks to Miss P. Davies for the help in preparation of this paper for publication and to Mrs. E. Slezakova for technical assistance.

### REFERENCES

AMIEL, J. L. AND MATHE, G.—(1960) *Experientia*, **16**, 563.
BECKER, F. F. AND GREEN, H.—(1960) *Expt. Cell. Res.*, **19**, 361.
BILLINGHAM, R. E. AND BRENT, L.—(1957) *Transpl. Bull.*, **4**, 67.
BUTLER, J. A. V., COHN, P. AND SIMSON, P.—(1960) *Biochim. biophys. Acta.*, **38**, 386.
DECARVALHO, S., RAND, H. J. AND MEYER, D. P.—(1960) *J. Lab. clin. Med.*, **55**, 706.
DANIELLI, J. F.—(1950) *Cold Spr. Harb. Symp. quant. Biol.*, **14**, 32.
DIMAYORCA, G. A., EDDY, B. E., STEWART, S. E., HUNTER, W. S., FRIEND, C. AND BENDICH, A.—(1959) *Proc. nat. Acad. Sci., Wash.*, **45**, 1805.
GRAFFI, A., FRITZ, D.—(1960) *Rev. franç. clin. biol.*, **5**, 388.
HLAVAYOVA, E.—(1957) *Neoplasma*, **4**, 10.
LACOUR, F., LACOUR, J., HAREL, J., HUPPERT, J.—(1960) *J. nat. Cancer Inst.*, **24**, 301.
LATARJET, R., REBEYROTTE, N., MOUSTACCHI, E.—(1959) *Haematologica Latina*, **2**, Suppl. II, 13.
LESLIE, E.—(1961) *Nature, Lond.*, **189**, 260.
LIEDER, N.—(1960) *Naturwissenschaften*, **47**, 308.
MEEK, E. S., HEWER, T. F.—(1959) *Brit. J. Cancer*, **13**, 121.
RIMAN, J., VESELY, J.—(1957) *Chem. Listy*, **51**, 1954.
SANDRITTER, W., FISCHER, H., SÜSSENBERGER, K., SCHIEMER, H. G.—(1959) *Exp. Cell Res.*, **17**, 197.
SETTERFIELD, G., NEELIN, J. M., NEELIN, E. M., BAYLEY, S. T.—(1960) *J. mol. Biol.*, **2**, 416.
SEVAG, M. G., LACKMAN, D. B., SMOLENS, J.—(1938) *J. biol. Chem.*, **124**, 425.
SIMONSEN, M.—(1957) *Acta path. microbiol. scand.*, **40**, 480.
STASNEY, J., CANTAROW, A., PASCHKIS, K. E.—(1950) *Cancer Res.*, **10**, 775.
STEDMAN, E., STEDMAN, E., PETTIGREW, F.—(1944) *Biochem. J.*, **38**, XXXI.
STOLK, A.—(1960) *Naturwissenschaften*, **47**, 88.
THIERY, C.—(1950) *C.R. Soc. Biol. Paris*, **144**, 745.
TOURTELLOTTE, W. W., STORER, J. B.—(1950) *Cancer Res.*, **10**, 783.
VALLADARES, V.—(1960) *Med. expt.*, **2**, 309.
WALLER, J. P., HARRIS, J. I.—(1961) *Proc. nat. Acad. Sci. Wash.*, **47**, 18.
WOODRUFF, M. F. A., SPARROW, M.—(1957) *Transpl. Bull.*, **4**, 157.
ZBARSKI, I. B., PEREVOZHCHIKOVA, K. A.—(1944) *Byull. eksp. biol. med.* (U.S.S.R.), **38**, 61.